# Evaluating the effectiveness of nonsteroidal anti‐inflammatory drug(s) for relief of pain associated with temporomandibular joint disorders: A systematic review

**DOI:** 10.1002/cre2.241

**Published:** 2019-08-21

**Authors:** Sachin Kulkarni, Samuel Thambar, Himanshu Arora

**Affiliations:** ^1^ School of Dentistry and Oral Health Griffith University Gold Coast QLD Australia; ^2^ Department of Oral and Maxillofacial Surgery Gold Coast University Hospital Southport QLD Australia

**Keywords:** NSAIDs, oral, pain, pharmacotherapy, temporomandibular joint disorders, topical

## Abstract

**Aim:**

The aim of this systematic review was to compile the latest evidence to assess the effectiveness of nonsteroidal anti‐inflammatory drug(s) (NSAID) in patients with temporomandibular joint disorders (TMDs) in relieving pain. TMDs are a group of musculoskeletal disorders that affect the temporomandibular joint and/or masticatory muscles.

**Methods:**

After a literature review, a comprehensive search was conducted via Pubmed, Scopus, Google Scholar, and Cochrane databases with a systematic search strategy. The inclusion criteria were randomised controlled trials in humans, published in the last 50 years evaluating the effect of NSAIDs on TMDs. Although this duration chosen would potentially identify studies that have utilised outdated treatments, research methodology, and TMDs diagnostic criteria, and this has been considered before making clinical recommendation, it was used to advise future methodological changes necessary. The included studies were subjected to full‐text review.

**Results:**

Out of 646 studies initially identified through searches, 12 were selected for full‐text review of which 11 were included in the data synthesis. All 11 studies were randomised controlled trials. In total, 424 patients were included in this review. The earliest study included was 1996. Diagnostic criteria varied among all studies, and some did not specify enough signs and symptoms to construct a diagnosis. Intervention varied among all studies, as did the control. Nonspecific diagnosis, variable control groups, and heterogenous intervention protocols affected the outcome of this review. Despite the reduction of pain in the joint and/or masticatory muscles as well as improved range of motion, conclusive clinical recommendation could not be made.

**Conclusion:**

Heterogeneity did not allow for definitive conclusion; however, there was some evidence to support the use of NSAIDs in patients with TMDs for relief of pain. Further studies with strict, consistent diagnostic criteria and treatment are required.

## 1 INTRODUCTION

Temporomandibular disorders (TMDs) are a group of conditions clinically presenting with common symptoms of pain, joint sounds, and restricted jaw movement. Each condition is defined clinically and often confirmed radiographically. The DC/TMD published in 2014 details the definition of individual conditions that form part of this cluster known as TMDs(Schiffman et al., 2014). They affect up to 12% of the population and pose a significant burden to the society, costing up to 4 billion dollars annually in United States alone(Schiffman et al., 2014). Treatment modalities range from conservative management to surgical. Up to 90% of the patients find relief in conservative management techniques. This includes occlusal splints, physical therapy, diet modification, and pharmacological agents(Lomas, Gurgenci, Jackson, & Campbell, 2018).

Various pharmacological agents are implicated in the management of TMDs. The different classes include nonsteroidal anti‐inflammatory drugs (NSAIDs), opioid analgesics, steroids, antianxiety agents, and muscle relaxants. A 2010 Cochrane review evaluated the efficacy of these pharmacological interventions inconclusively(Lele & Hooper, 2004).

### 1.1 Rationale for management of TMDs

Management goals for patients are in line with those for other musculoskeletaldisorders: reduction in experience of pain, restoration of function, and reduction in interferences with daily activities (de Leeuw & Klasser, 2018; Schiffman et al., 2014). Although evidence suggests that 90% of patients show few or no symptoms after 7 years of conservative treatment (physical therapy, behavioural modification, medications, and orthopaedic appliances), there continues to be confusion among clinicians on the choice of treatment(de Leeuw & Klasser, 2018).

The following factors have been associated with development of TMDs; however, no single cause has been found. Trauma has been defined as direct (to the mandible or Temporomandibular joint (TMJ)) possibly causing structural failure, indirect through flexion–extension injury (though controversial), and microtrauma hypothesised to be from sustained loading in masticatory muscles through posture or parafunctional habits. Both indirect and microtrauma have insufficient evidence to support their aetiology. Traditional cause of TMDs has been viewed to be occlusal variation; however, only loss of posterior support and unilateral crossbites have shown some association(de Leeuw & Klasser, 2018).

Pathophysiologic morphological changes, whether as a result of osteoarthritis or not, have been found in TMJ, like other joints in the body. Disc derangement with or without reduction is known to occur commonly with or without symptoms. Osteoarthritic changes due to disc derangement with reduction tend to appear after many years and, therefore, may not necessarily benefit for immediate irreversible treatment options. Psychosocial factors that have been found to have an association are clenching, where prolonged clenching might have initiate or advance TMD symptoms, and stress‐related disorders including anxiety. Incidence of anxiety has been found to be higher in individuals with TMDs and is known to relate to altered pain perception and tolerance(de Leeuw & Klasser, 2018).

Therefore, management of patients with TMDs will require an assessment of underlying aetiology and targeted management technique including referral to appropriate specialist. Techniques commonly used are restoring posterior vertical dimension, mandibular advancement splint with some evidence for disc displacement without reduction, psychosocial assessment and referral to psychologist/counsellor for stress‐management techniques, biofeedback for habit awareness, and treatment of acute inflammatory conditions with NSAIDs(de Leeuw & Klasser, 2018; Lele & Hooper, 2004). Frequently, multiple techniques are used together due to lack of clear identification of aetiologies or identification of multiple underlying cause of symptoms.

### 1.2 Background on NSAIDs

Roughly in the 400 BC, Hippocrates was known to prescribe an extract from the bark of a willow tree for treatment of inflammation. However, it was not until the 17th century that the active ingredient salicin was discovered. Later, Bayer in 1899 developed a more palatable form—acetylsalicylic acid (aspirin). The mechanism of action would remain unclear up until the 1970s, when John Vane was credited to discovering it. This has allowed decades of novel NSAID production and use in treating various inflammatory conditions(de Leeuw & Klasser, 2018).

The main mechanism of action is inhibition of the enzyme prostaglandin H synthase or cyclooxygenase (COX 1 and COX 2). The COX pathway produces prostanoids that have been known to play an important role in the mediation of inflammation and pain. This placed NSAIDs in the limelight for the treatment of inflammatory pain such as that present in TMDs. However, over time, various side effects have been identified. The most frequently reported gastrointestinal (GI) effects are due to the inhibition of gastroprotective prostanoid production via COX 1. Symptoms that patients often experience are heartburn and GI ulcer formation. Prostanoids are also known to modulate normal renal function via maintaining vascular tone and normal blood flow. Reversible or irreversible inhibition of COX 1 in platelets blocks thromboxane A2 resulting in reduced risk of thrombosis but a greater risk of bleeding(de Leeuw & Klasser, 2018).

Despite the risks, inconclusive evidence, and availability of other pharmacological agents, NSAIDs remain a common prescription for pain‐related TMDs. Therefore, the aim of this systematic review is to critically review the existing literature and evaluate the null hypothesis that NSAIDs does not produce any difference in pain and mouth opening in patients with TMDs. The specific question was constructed using the participants, interventions, comparators, outcomes, and study design format as per the Preferred Reporting Items for Systematic Reviews and Meta‐Analyses checklist(Moher et al., 2009): Does NSAIDs (oral/topical) reduce pain and improve mouth opening in patients with temporomandibular joint disorder(s)? The participants include humans diagnosed with temporomandibular joint disorder(s). The intervention group (NSAIDs) was compared with that of the specified control group (other conservative management techniques including laser or placebo or other pharmacological therapy), the primary outcome measured was pain (on a validated pain scale that must be specified), and secondary outcome was mouth opening (maximum assisted/unassisted).

## 2 METHODS

The Preferred Reporting Items for Systematic Reviews and Meta‐Analyses checklist has been followed while conducting this systematic review(Moher et al., 2009). A comprehensive search of PubMed, Scopus, Google Scholar, and Cochrane library was conducted by S. K. and S. T. with the following search strategy: “anti‐inflammatory agents, non‐steroidal” AND (“myalgia” or “bruxism” OR “temporo‐mandibular joint disorders”). The search covered studies published up until April 2018, and additional sources were identified from the references.

Both authors added the articles to the citation manager EndNote X8 ® (Clarivate Analytics, New York City), and duplicates were eliminated. They were then screened by both the authors (S. K. and S. T.) for title and abstracts to sort articles into an excluded, included, and unsure folder. Third author (H. A.) was consulted when agreement could not be reached. The inclusion criteria applied were (a) study was published in the last 50 years in a professional or scientific English journal; (b) study was an in vivo (human) experiment; and (c) study is a randomised controlled trial (RCT). The exclusion criteria were (a) in vitro studies involving cells and tissues, not whole animals; and (b) studies with less than 10 subjects.

**Figure 1:**
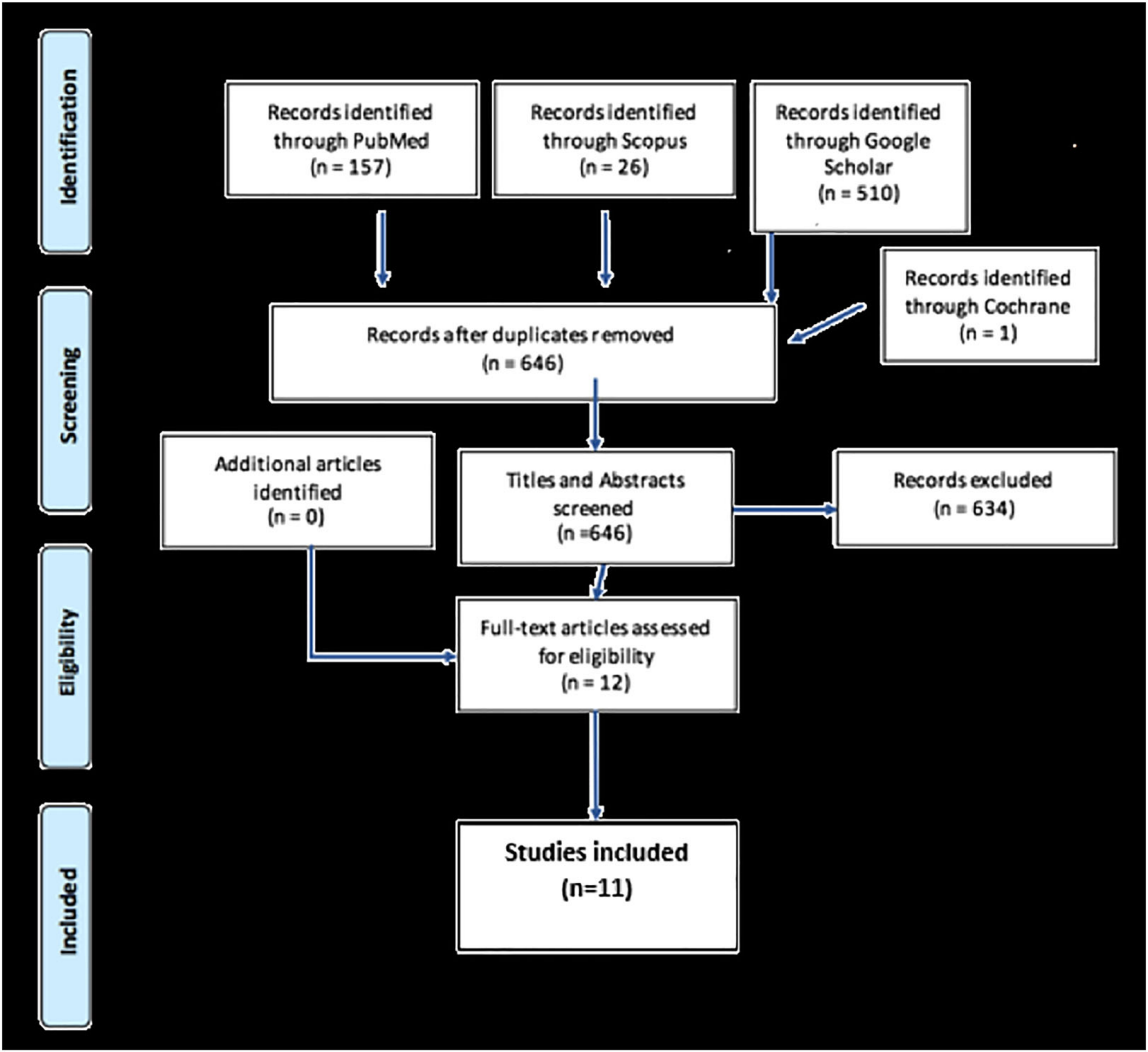
PRISMA flow chart demonstrating the selection process

The selection process can be seen in Figure 1.

### 2.1 Study outcomes

1. Pain measured on a validated pain scale
2. Mouth opening (assisted/unassisted maximum)

### 2.2 Risk of bias

The methodological quality of human studies was assessed using the JADAD scale, a validated scale that includes assessment of blinding and randomisation(Halpern & Douglas, 2005). This was done independently by S. K. and S. T., where a score of 3 or higher was determined to be of high quality, unless particular problems were found that would reduce their quality (Table 1). A consensus was reached after discussion between the two authors and validated by the third author H. A. All studies were randomised, with majority of them also having double or triple blinding (de Carli et al., 2013; Ekberg et al., 1996; Kurita Varoli et al., 2015; Lobo et al., 2004; Marini et al., 2012; Singer & Dionne, 1997; Ta & Dionne, 2004; Thie et al., 2001). Two articles did not mention blinding(Di Rienzo Businco et al., 2004; Yuasa & Kurita, 2001). One of these studies reported having a control group that was randomly selected; however, whether the control group was informed of their treatment is not reported. As the outcome measure of this study included self‐reporting on visual analogue scale (VAS) for joint pain at rest and upon functioning, as well as interference with daily life, this has been assumed to impact the reported VAS scores(Yuasa & Kurita, 2001) exaggerating the difference between treatment and control group outcome. One article was a single blinded trial (Mejersjo & Wenneberg, 2008). The median JADAD score was 5, with only two studies scoring 3^14,15^ and one study scoring 4 as it was a single‐blinded trial(Mejersjo & Wenneberg, 2008). One study was funded by the firm that manufactures the tested topical cream Theraflex‐TMJ^12^. Although experimental design was of high quality, the funding could have influenced the analysis, interpretation, and sharing of data. Therefore, these four studies have been assessed as being moderate in quality(Di Rienzo Businco et al., 2004; Lobo et al., 2004; Mejersjo & Wenneberg, 2008; Yuasa & Kurita, 2001), and all other studies have been assessed as high quality.

**Table 1:** Risk of bias assessed with JADAD scale

Author(s)	Is randomisation mentioned?	Is method of randomisation appropriate?	Is blinding mentioned?	Is method of blinding appropriate?	Is fate of all participants known?	Score
de Carli et al. (2013)	Yes	Yes	Yes	Yes	Yes	5
Marini, Bartolucci, Bortolotti, Gatto, and Bonetti (2012)	Yes	Yes	Yes	Yes	Yes	5
Mejersjo and Wenneberg (2008)	Yes	Yes	Yes	No. Single‐blinded trial.	Yes	4
Ta and Dionne (2004)	Yes	Yes	Yes	Yes	Yes	5
Thie, Prasad, and Major (2001)	Yes	Yes	Yes	Yes	Yes	5
Yuasa and Kurita (2001)	Yes	Yes	No	N/A	Yes	3
Di Rienzo Businco, Di Rienzo Businco, D'Emilia, Lauriello, and Coen Tirelli (2004)	Yes	Yes	No	N/A	Yes	3
Ekberg, Kopp, and Akerman (1996)	Yes	Yes	Yes	Yes	Yes	5
Kurita Varoli et al. (2015)	Yes	Yes	Yes	Yes	Yes	5
Lobo et al. (2004)	Yes	Yes	Yes	Yes	Yes	5
Singer and Dionne (1997)	Yes	Yes	Yes	Yes	Yes	5

## 3 RESULTS

A total of 646 articles were identified from search of various databases. After screening for abstracts and titles, 12 articles were selected for full‐text review. All 12 articles selected are RCTs. They all fulfilled the inclusion criteria, but 11 were included in synthesis, as full‐text article for one could not be found through all three databases, and an attempt to contact the authors was made for full text with no response. A total of 424 patients were included. The smallest study had 18 participants and largest had 68(Kurita Varoli et al., 2015; Ta & Dionne, 2004). The oldest article included was published in 1996(Ekberg et al., 1996).

### 3.1 Intervention and control

Various NSAIDs have been utilised in the studies ranging from topical diclofenac to piroxicam, palmitolyethanolamine, ibuprofen, and selective COX 2 inhibitor celecoxib (de Carli et al., 2013; Di Rienzo Businco et al., 2004; Marini et al., 2012). Glucosamine succinate and diazepam has also been compared with that of NSAIDs in two studies(Di Rienzo Businco et al., 2004; Singer & Dionne, 1997; Thie et al., 2001). One study also assessed an over‐the‐counter product called Theraflex‐TMJ^12^. This formulation contained methyl salicylate (related compound to acetylsalicylic acid), copper pyrocarboxylate, and zinc pyrocarboxylate, with unreported concentrations. It has been reported in literature that mechanism of anti‐inflammatory action of copper is due to its ability to directly reduce the production of free radicals, inactivation of them, and prevention of release of lysosomal enzymes(Beveridge, 1998). Zinc has also been implicated in antioxidant activity and has also been shown to restrict immune activation(Jarosz, Olbert, Wyszogrodzka, Młyniec, & Librowski, 2017). Adjunctive therapy with laser, physical therapy, and occlusal splints have also been studied(de Carli et al., 2013; Kurita Varoli et al., 2015). Dosages have not been specified in some articles(Kurita Varoli et al., 2015; Mejersjo & Wenneberg, 2008; Singer & Dionne, 1997). The general characteristics of studies as well as interventions and control have been specified in Table 2. As noted, the control groups varied from no treatment, to alternative pharmaceutical agents, to occlusal splints. Separate control groups, and different interventions, including some nonreported dosages, meant quantitative analysis would not yield clinically relevant result.

**Table 2:** General characteristics of included studies

Author(s)	Type of study	JADAD score	No. of participants	Diagnosis according to DC/TMD	Observation period	Intervention	Control
de Carli et al. (2013)	Randomised control trial	5	32	Arthralgia (ICD‐9 524.62; ICD‐10 M26.62)	30 days	Infrared laser therapy on 10 points around TMJ and muscles, for four sessions. AND one capsule a day of piroxicam 20 mg during 10 days.	Placebo piroxicam and placebo laser
Marini et al. (2012)	Randomised control trial	5	24	Arthralgia (ICD‐9 524.62; ICD‐10 M26.62) and degenerative joint disease (ICD‐9 715.18; ICD‐10 M19.91)	2 weeks	Palmitoylethanolamine (PEA) 300 mg in the morning and 600 mg in the evening for 7 days and 300 mg twice a day for seven more days	Ibuprofen 600 mg three times a day for 2 weeks
Mejersjo and Wenneberg (2008)	Randomised control trial	4	29	Arthralgia (ICD‐9 524.62; ICD‐10 M26.62) and degenerative joint disease (ICD‐9 715.18; ICD‐10 M19.91)	1 year follow‐up	Diclofenac sodium	Occlusal splint therapy
Ta and Dionne (2004)	Randomised control trial	5	68	Arthralgia (ICD‐9 524.62; ICD‐10 M26.62) and disc displacement with reduction (ICD‐9 524.63; ICD‐10 M26.63)	6 weeks	Celecoxib 100 mg twice a day or naproxen 500 mg twice a day for 6 weeks	Placebo for 6 weeks
Thie et al. (2001)	Randomised control trial	5	39 (34)	Arthralgia (ICD‐9 524.62; ICD‐10 M26.62) and degenerative joint disease (ICD‐9 715.18; ICD‐10 M19.91)	90 days	Glucosamine succinate 500‐mg TID	Ibuprofen 400‐mg TID
Yuasa and Kurita (2001)	Randomised control trial	3	60	Disc displacement without reduction with limited opening (ICD‐9 524.63; ICD‐10 M26.63)	2 and 4 weeks	NSAID and physical therapy	Untreated
Di Rienzo Businco et al. (2004)	Randomised control trial	3	36	Lack of reported symptoms for classification	14 days	Topical diclofenac sodium 16 mg/ml 10 drops four times a day for 14 days	Oral diclofenac 50 mg twice a day for 14 days
Ekberg et al. (1996)	Randomised control trial	5	32	Lack of reported symptoms for classification	—	Diclofenac sodium 50 mg 2/3 times a day	Placebo
Kurita Varoli et al. (2015)	Randomised control trial	5	18	Myalgia—lack of reported symptoms for specific and associated classification	10 days	Occlusal splint with sodium diclofenac and occlusal splint with panacea (diclofenac + carisoprodol + acetaminophen)	Occlusal splint with placeboe
Lobo et al. (2004)	Randomised control trial	5	52	Arthralgia (ICD‐9 524.62; ICD‐10 M26.62) and Myalgia—not specified due to lack of adequate description of muscle assessment and pain	2 weeks	Topical Theraflex‐TMJ twice daily for 2 weeks	Placebo cream
Singer and Dionne (1997)	Randomised control trial	5	39	Myalgia—lack of reported symptoms for specific and associated classification	2 and 4 weeks	Diazepam, ibuprofen 2,400 mg per day, and a combination	Placebo

Abbreviation: TMD, temporomandibular joint disorder.

### 3.2 Effect of NSAIDs

Table 3 compiles the outcome measures of all studies, as well as relevant data regarding the effect of NSAIDs on two outcome measures—change in pain on VAS of 0‐100 and change in maximum mouth opening (mm). Majority of the studies utilised self‐reporting of pain scores, whether at rest or upon mandibular motion, as well as measurement of tenderness to palpation, and measure of interincisal distance for maximum mouth opening, whether pain free or with pain.

**Table 3:** Pain scale and outcome with regard to NSAIDs treatment

Author(s)	Outcome measures(s)	Average change in pain score (0–100) [%change from baseline]^a^	Average change in mouth opening (mm)	Comments
de Carli et al. (2013)	Visual analogue scale 0–100 and maximum opening (mm)	Laser with NSAID: −19.36 (*P* < .0001) [64.5%] NSAID alone: −25.8 (*P* < .0001) [79.1%]	Laser with NSAID: +0.86 (*P* = .4273) NSAID alone: +1.17 (*P* = .1735)	VAS recording upon palpation or spontaneous pain not differentiated in the study
Marini et al. (2012)	Visual analogue scale 0–100 (self‐reported) and maximum opening (mm)	NSAID: −31.00 [45.3%]	NSAID: +2.13	No statistical analysis of change in the same group of NSAID and PEA done. Statistical difference between PEA and NSAID conducted alone, with PEA giving significant higher amount of reduction in pain
Mejersjo and Wenneberg (2008)	Visual analogue scale 0–100 and maximum opening (mm)	On movement: −62.00 (*P* < .01) [66.7%] On palpation: −42.00 (*P* < .01) [84.0%]	Maximum: +6.00 (*P* < .01)	Have not mentioned if the mouth opening measured was with pain or pain free
Ta and Dionne (2004)	Visual analogue scale 0–100 and maximum opening (mm)	Celecoxib: −21.08 (*P* < .01) [42.2%] Naproxen: −33.05 (*P* < .01) [73.8%]	Celecoxib:+8.22 (*P* < .01) Naproxen: +12.5 (*P* < .01)	
Thie et al. (2001)	Visual analogue scale 1–100 and maximum opening (mm)	On movement: −5.93 (*P* < .001) [26.2%] On palpation: −4.33 (*P* < .001) [55.4%]	Pain free: +8.39 (*P* < .001) Voluntary: +4.06 (*P* < .001)	
Yuasa and Kurita (2001)	Visual analogue scale 0–100 and maximum opening (mm)	On movement: −23.5 (*P* < .001) [45.6%]	Maximum: +8.5 (*P* < .001)	Patient's with more severe TMJ dysfunction scores responded better to treatment. Have not mentioned if the mouth opening measured was with pain or pain free
Di Rienzo Businco et al. (2004)	Visual analogue scale 1–10 (self‐reported)^b^ and maximum opening (mm)	Topical NSAID: −61.0 [84.7%] Oral NSAID: −59.0 [83.1%]	Maximum on a VAS (0–5 where five is maximum opening and zero is difficulty opening): ‐ Topical NSAID: +0.7 ‐ Oral NSAID: +1.0	Nil significant difference between oral and topical NSAID found. No statistical analysis within‐group pre‐ and post‐op done. Nil data on actual measurement of mouth opening
Ekberg et al. (1996)	Visual analogue scale 0–100	Statistically significant reduction in frequency of pain No statistically significant difference between placebo and NSAID for severity of pain Tenderness to palpation of muscles had statistically significant greater reduction than placebo	No statistically significant difference between placebo and NSAID	No VAS values reported, although reported that they were measured. It is reported that the diagnosis and assessment did not include assessment of noninflammatory origin of pain, clouding the reliability of results
Kurita Varoli et al. (2015)	Visual analogue scale 0–10	Actual values not reported. However, change in both NSAID (one diclofenac and two was a combination with acetaminophen, diclofenac, carisoprodol, and caffeine). Statistically significant difference in VAS for both formulas reported; however, no difference between placebo and the two formula groups	Mouth opening not assessed	All patients received occlusal splint therapy, including the placebo group. Therefore, the difference between NSAID and placebo group was not a reliable mode of comparison. It is therefore found that occlusal splint therapy also reduced VAS scores but NSAID treatment did not significantly improve the reduction
Lobo et al. (2004)	Numerical graphic rating scale (NGRS) 0–10 point scale	−1.26, i.e., 12.6% reduction (*P* < .01 but >.001)	Mouth opening not assessed	Theraflex‐TMJ contains methyl salicylate, copper pyrocarboxylate, and zinc pyrocarboxylate
Singer and Dionne (1997)	Visual analogue scale 1–100, McGill pain questionnaire, and maximum opening (mm)	Ibuprofen alone: −2.2 (*P* < .05) [14.2%] Ibuprofen and diazepam combination: −2.1 (*P* < .05) [16.4%]	Statistically insignificant results. Ibuprofen alone: +1.7 mm Ibuprofen and diazepam combination: −0.7 mm	

Abbreviations: NSAIDs, nonsteroidal anti‐inflammatory drugs; VAS, visual analogue scale.

a %Change has been calculated as per follows: [Final score − initial score]/initial score × 100.

b The VAS scores have been adjusted to 0–100, although not exactly representative, this allowed for comparison between studies.

#### 3.2.1 Pain scores

One study utilised Numerical Graphic Rating Scale 0–10, which is also a validated pain scale for head and neck pain(Lobo et al., 2004). In two studies(Ekberg et al., 1996; Kurita Varoli et al., 2015), no VAS scores were reported although there was positive result for pain scores in both studies. All studies reported a reduction in pain scores and were statistically significant. *P* values and change in pain score from baseline have been reported in Table 3. Percentage change from baseline was also calculated, and the average %change from baseline in pain scores on palpation due to NSAID treatment alone was 54.84%. Most studies consistently reported changes of over 30%, except two(Lobo et al., 2004; Thie et al., 2001). This has also been reported in Table 3.

#### 3.2.2 Maximum mouth opening

Two studies did not assess mouth opening(Kurita Varoli et al., 2015; Lobo et al., 2004); however, rest of the studies assessed it as interincisal distance. Only one studiy(Thie et al., 2001) specified pain‐free and assisted opening, and the rest did not specify if the assessment was pain free, voluntary, or passive. In all cases of assessment, positive increase in mouth opening has been reported.

### 3.3 TMD diagnosis

Diagnoses studied ranged from arthralgia, osteoarthritis, pain secondary to disc displacement with reduction, pain secondary to disc displacement without reduction, myogenous facial pain to masticatory muscle pain blinding(de Carli et al., 2013; Ekberg et al., 1996; Kurita Varoli et al., 2015; Lobo et al., 2004; Marini et al., 2012; Singer & Dionne, 1997; Ta & Dionne, 2004; Thie et al., 2001; Yuasa & Kurita, 2001). One study did not specify a diagnosis, although it was reported to be a dysfunction of TMJ^15^. In order to eliminate the heterogenous diagnostic criteria, the task to convert the diagnosis to a standardised DC/TMD(Schiffman et al., 2014) was conducted, as represented in Table 4. Six studies were adjusted to arthralgia (DC/TMD)(de Carli et al., 2013; Lobo et al., 2004; Marini et al., 2012; Mejersjo & Wenneberg, 2008; Ta & Dionne, 2004; Thie et al., 2001), three of which also had degenerative joint disease concurrently (Marini et al., 2012; Mejersjo & Wenneberg, 2008; Thie et al., 2001). One of those six studies also had concurrent disc displacement with reduction(Ta & Dionne, 2004). Another study specified some inconclusive pain symptoms and concluded diagnosis as disc displacement without reduction with limited opening and no pain diagnosis of arthralgia or myalgia(Yuasa & Kurita, 2001). There were four other studies with incomplete description or assessment of pain that resulted in not being able to adjust the diagnostic category and one study(Lobo et al., 2004) where specific muscle pain diagnosis could not be achieved (Di Rienzo Businco et al., 2004; Ekberg et al., 1996; Kurita Varoli et al., 2015; Lobo et al., 2004; Singer & Dionne, 1997).

**Table 4:** Diagnostic category adjustments

Author(s)	Signs and symptoms	Duration of symptoms	Diagnosis/diagnostic criteria	Diagnosis according to DC/TMD
de Carli et al. (2013)	• Pain in one or both joint sites (lateral pole and/or posterior attachment) during palpation; plus • One or more of the following self‐reports of pain: pain in the region of the joint, pain in the joint during maximum unassisted opening, pain in the joint during assisted opening, and pain in the joint during lateral excursion • Absence of coarse crepitus • Tender points on palpation of posterior bilaminar zone, posterior aspect of TMJ capsule, masseter (superior, middle, and inferior), and temporal (anterior, middle, and posterior)—excluded from study groups to only include arthralgia alone	Not specified	Arthralgia of TMJ–RDC/TMD	Arthralgia (ICD‐9 524.62; ICD‐10 M26.62)
Marini et al. (2012)	• Pain in one or both joints at rest and during function • Evoked pain on TMJ palpation • Crepitus • Patients with musculoskeletal pain, myogenic pain, depressive disorders, odontogenic pain, pregnancy, malignancy, and other systemic rheumatological diseases excluded • Radiographic assessment for anatomical changes of both hard and soft tissues—flattening and erosion of the articular surface	Not specified	Separate diagnoses for patients were not specified. Included patients were diagnosed with either both or one of Osteoarthritis of TMJ and arthralgia of TMJ–RDC/TMD	Arthralgia (ICD‐9 524.62; ICD‐10 M26.62) and degenerative joint disease (ICD‐9 715.18; ICD‐10 M19.91)
Mejersjo and Wenneberg (2008)	• Self‐reported TMJ pain • Tenderness to palpation lateral and/or posterior of TMJ • Pain in TMJ on mandibular movement • Coarse crepitus and/or radiological signs of erosions and/or sclerosis of cortical outline, flattening of joint surfaces, and/or osteophyte formation • Nil mention if pain is acute or chronic	Chronic	Osteoarthritis of TMJ–RDC/TMD	Arthralgia (ICD‐9 524.62; ICD‐10 M26.62) and degenerative joint disease (ICD‐9 715.18; ICD‐10 M19.91)
Ta and Dionne (2004)	• Joint pain at rest • Evoked pain on palpation of TMJ • TMJ reduction consists of joint reciprocal clicking or joint noise with mandibular movement • Patients with myogenic pain only included if secondary to their Disc displacement with reduction and arthralgia of TMJ • Disc displacement with reduction confirmed with MRI • Minnesota multiphasic personality inventory‐2 used to exclude patient with severe personality or psychosis disorders • On average TMD pain duration was 3.1 years (classified as chronic). Minimum duration of page was 2.4 months and maximum 180 months • Nil mention of limitation in mouth opening		Disc displacement with reduction and arthralgia of TMJ–RDC/TMD	Arthralgia (ICD‐9 524.62; ICD‐10 M26.62) and disc displacement with reduction (ICD‐9 524.63; ICD‐10 M26.63)
Thie et al. (2001)	• Moderate (VAS minimum of 3 on VAS of 0–10) pain of TMJ upon function (chewing, yawning, talking, and laughing) • Radiographic evidence of degenerative joint disease (subchondral sclerosis, osteophytic formation, erosion, and joint space narrowing) • Nil mention if pain is acute or chronic	Not specified	Osteoarthritis of TMJ–American board of orofacial pain diagnostic criteria	Arthralgia (ICD‐9 524.62; ICD‐10 M26.62) and degenerative joint disease (ICD‐9 715.18; ICD‐10 M19.91)
Yuasa and Kurita (2001)	• Disc displacement without reduction and without osseous changes (confirmed by MRI) • Moderate to severe pain (minimum score of 30 on VAS of 0–100) in TMJ alone at rest, with motion, on chewing and interference with daily life (any patient with pain in region other than TMJ were excluded) • Limited mouth opening (ranging from • Closed lock • Nil mention if pain is acute or chronic	Not specified	TMJ dysfunction—disc displacement without reduction and without osseous changes—nil diagnostic criteria mentioned	Disc displacement without reduction with limited opening (ICD‐9 524.63; ICD‐10 M26.63)
Di Rienzo Businco et al. (2004)	• Pain in ear and mandibular region • Limited mouth opening upon a VAS (0–5 where 5 is the maximum functional opening) • Nil mention if pain is acute or chronic • Nil other symptoms and signs reportedly assessed • Only some patients underwent stratigraphy “when needed”—nil criteria for the necessity reported. Nil report of results from stratigraphy	Not specified	Craniomandibular dysfunction—no mention of diagnostic criteria	Lack of reported symptoms for classification
Ekberg et al. (1996)	• Pain localised to TMJ for a minimum of 6 weeks (on average 11.5 months, classed as chronic pain) • Lateral or posterior tenderness to the TMJ • Spontaneous pain of TMJ • Pain on yawning and chewing in TMJ • Muscle pain not described specifically • Joint sounds not described specifically • Limitation in opening not described specifically	Chronic	Temporomandibular joint pain—no mention of diagnostic criteria	Lack of reported symptoms for classification
Kurita Varoli et al. (2015)	• Provoked pain in masseter, temporalis, sternocleidomastoid, and trapezius • Provoked pain upon palpation of TMJ lateral pole • Specifics of nature of pain—localised versus referred, exact location of palpation, force of palpation, and palpation of other muscles for exclusion not mentioned • Limitation in mouth opening not mentioned • No evaluation of TMJ sounds mentioned	Chronic	Masticatory muscle pain—no mention of diagnostic criteria	Myalgia—lack of reported symptoms for specific and associated classification
Lobo et al. (2004)	• Reported pain in masseter muscle either at rest or during function • Pain on palpation of masseter muscle • Pain in TMJ either at rest or during function • Specifics of nature of pain—localised versus referred, exact location of palpation, force of palpation, and palpation of other muscles for exclusion not mentioned	Not specified	Arthralgia of TMJ–RDC/TMD	Arthralgia (ICD‐9 524.62; ICD‐10 M26.62) and myalgia—not specified due to lack of adequate description of muscle assessment and pain
Singer and Dionne (1997)	• Pain of at least three month duration • Muscle tenderness in muscles of mastication • Limited opening and presence of clicking in some patients, although not a necessary inclusion criteria • Exclusion criteria: clinical or radiographic evidence of TMJ crepitus, tenderness on palpation through external acoustic meatus, and erosion of condyle	Chronic	Muscle pain—no mention of diagnostic criteria	Myalgia—lack of reported symptoms for specific and associated classification

Abbreviations: TMD, temporomandibular joint disorder; VAS, visual analogue scale.

### 3.4 Side effects

Topical Theraflex‐TMJ and topical diclofenac caused skin irritation in two studies that were temporary(Di Rienzo Businco et al., 2004; Lobo et al., 2004). GI side effects of oral diclofenac have also been noted(Di Rienzo Businco et al., 2004). This is consistent with current evidence for the use of oral and topical NSAIDs(Klinge & Sawyer, 2013).

## 4 DISCUSSION

The aim of a systematic review is to synthesise results from primary studies, critically appraise them, and eventually support evidence‐based practice. In order to provide a conclusion that can be applied, it is imperative to have synthesis of data, which due to heterogeneity became impossible. In the present systematic review, there was heterogeneity in the type of intervention, dosage of NSAID used, control used, duration of study, and the diagnosis of TMD being treated. Earliest study included was published in 1996, and there is evidence to suggest that NSAIDs have been further researched, with a particular focus on reducing side effects specifically GI and cardiovascular. Clinical recommendations by the American Heart Association and American College of Rheumatology suggest a detailed history and assessment of cardiovascular risk factors (such as history of cardiovascular disease, recent bypass surgery, oedema, hypercholesterolemia, hypertension, and angina), as well as history of gastric ulcers and bleeding. Management of patients with history or higher likelihood of developing gastric ulcers can be done with a combination therapy of COX 2 selective NSAIDs or NSAIDs with proton‐pump inhibitor, provided contraindications and co‐morbidities are assessed. However, in all cases, the recommendation remains that the lowest effective dose for the shortest duration of time be used(Conoghan, 2012).

### 4.1 Diagnosis

A consistent, easily usable, reliable, and valid diagnostic criteria are required for both clinical and research applications. Clinicians appreciate the ease with which they can communicate among each other, as well as to the patients. Researchers appreciate being able to compare, synthesise, and translate the same clinical question across various studies. With this understanding, the new dual‐axis diagnostic criteria for TMD (DC/TMD) were developed and updated in 2015. With growing understanding of TMD symptoms, it has become important to assess the behavioural aspects of pain‐related TMD, and this forms the second axis of diagnostic criteria(Schiffman et al., 2014).

Majority of the articles that have been included in this systematic review have been published before the introduction of this updated criteria (de Carli et al., 2013; Di Rienzo Businco et al., 2004; Ekberg et al., 1996; Kurita Varoli et al., 2015; Lobo et al., 2004; Marini et al., 2012; Singer & Dionne, 1997; Ta & Dionne, 2004; Yuasa & Kurita, 2001). The nomenclature, taxonomy, and validity of the diagnosis are not readily translated, although an effort was made to adjust the diagnoses to the current DC/TMD (Table 4), hence, introducing heterogeneity among all the RCTs. This has two implications. First, heterogenous diagnostic categories would mean that the effectiveness of NSAIDs cannot be concluded for individual categories and the specific treatment recommendation cannot be made. Second, future research implication would be to conduct a series of trials with same diagnostic categories to assess the effectiveness of NSAIDs that are also standardised in route of administration, application duration, type of NSAID, pain assessment technique, and pain reporting methods.

### 4.2 Pain scores

For any particular intervention to be effective in reducing pain, a reduction of roughly 30% from baseline is expected on VAS(Hawker, Mian, Kendzerska, & French, 2011). Despite achieving this on average (54.84%), there are three main criticisms with assessment of pain. First, differentiation between pain at rest and pain upon motion was not made, hence not allowing for direct comparison of scores. Second, pain assessment on palpation (with pressure that can vary between patients and between appointments) was subjective, and the use of algometer may allow to better quantify tenderness(Więckiewicz, Woźniak, Piątkowska, Szyszka‐Sommerfeld, & Lipski, 2015). Third, the diagnostic categories were mixed—especially acute and chronic pain.

VAS has been shown to be an effective measuring tool for acute pain in adults. However, chronic pain in adults has been unsatisfactorily measured by VAS. VAS has also been criticised as being used as a unidimensional measure of pain intensity, and not capturing the complex experience of chronic pain(Hawker et al., 2011).

Chronic pain has been known to differ from acute pain in multiple ways—there is a significant impact on work and daily functioning; relief of inflammation in the tissue may not relieve the patient of pain under central sensitisation theory; depression, anxiety, and prolonged negative feelings are more common; and reduced compliance to treatment. And although positive outcome has been obtained in this systematic review, and there is some evidence of benefit from NSAID treatment in chronic pain, it only forms a part of a larger care plan in a chronic pain patient(Ho et al., 2018).

### 4.3 TMDs as inflammatory conditions

The broad categories of TMDs include joint disorders and muscle disorders. Joint disorders include internal derangement of joint with disc displacements with/without reduction, with/without limited opening, and arthritic changes. Muscle disorders are classified as myalgia, myofascial pain, and myofascial pain with referral(Schiffman et al., 2014). Both categories exhibit inflammation. Joint disorders such as osteoarthritis or rheumatoid arthritis, as well as retrodiscitis, are inflammatory conditions that have been known to respond to NSAIDs. Retrodiscitis has been defined as the inflammation of the retrodiscal lamina that is present posterior to the disc between the mandibular condyle and the glenoid fossa of the temporal bone (Kim, 2014).

Muscular disorders have characteristic inflammation in muscles with pain either located locally in the masticatory muscles or referred to regions around it. Both the joint disorders and muscular disorders hence benefit from NSAID treatment(Kim, 2014). There are only two studies that have assessed stand‐alone NSAID treatment with placebo but cannot be compared due to lack of specific diagnosis(Di Rienzo Businco et al., 2004; Ta & Dionne, 2004). Based on current evidence, NSAIDs individually show positive outcomes; however, there is lack of sufficient studies, clear diagnosis, and clear identification of aetiologies in the patients that have been studied. All other studies have used it in conjunction with other treatments such as occlusal splints, lasers, and other pharmacological agents. This prevented assessment of efficacy of individual techniques. However, positive results were obtained with NSAIDs being used as an adjunct.

### 4.4 NSAID route of administration

Only two articles used topical route of administration for pharmacological agent. Topical diclofenac 15 mg/ml (four times a day for 14 days) was compared with oral diclofenac 50 mg (twice a day for 14 days) with no statistically significant difference in their effectiveness in reducing self‐reported pain and improvement in function of TMJ (Di Rienzo Businco et al., 2004; Lobo et al., 2004). The results from these studies are consistent with a comprehensive review published in 2013. It is found among 600 subjects that topical NSAIDs provided similar efficacy for both acute and chronic injury when compared with oral NSAIDs.

## 5 CONCLUSION

This systematic review presented some discussion on current management goals and aetiologies and, more importantly, on effectiveness of NSAIDs in treatment of TMDs. There is some evidence to suggest that NSAIDs can alleviate pain and improve mouth opening in patients with TMDs. However, there is insufficient evidence to conclude the type, dosage, and duration of NSAID, for what diagnostic category of TMDs. There is inconsistency in the methodology between the primary studies making definitive conclusion impossible.

### 5.1 Implication for research

Further research is needed with consistent diagnostic criteria, NSAIDs studied as primary treatment as well as an adjunct therapy, and control groups are advised to be placebo. Distinction between acute and chronic pain, more specific signs and symptoms in regard to muscle groups involved, and psychosocial examination needs to be conducted and reported.

### 5.2 Implication for clinical practice

Psychologic factors need to be evaluated in conjunction with physical findings to confirm the diagnosis and to construct a list of possible aetiology to assist in choosing one or more management techniques. Topical route of administration may be preferred as limited evidence shows similar effectiveness as oral NSAIDs, with no GI side effects. Without further evidence, conclusion cannot be reached whether NSAIDs can be used individually or as an adjunct. However, in the management of TMDs, NSAIDs have some evidence of effectiveness.

## CONFLICT OF INTEREST

The authors declare that there is no conflict of interest regarding the publication of this article.

## FUNDING INFORMATION

No source of funding was utilised for this article.
